# YdjC chitooligosaccharide deacetylase homolog induces keratin reorganization in lung cancer cells: involvement of interaction between YDJC and CDC16

**DOI:** 10.18632/oncotarget.25145

**Published:** 2018-05-01

**Authors:** Eun Ji Kim, Mi Kyung Park, Hyun Jung Byun, Gyeoung Jin Kang, Lu Yu, Hyun Ji Kim, Jae Gal Shim, Ho Lee, Chang Hoon Lee

**Affiliations:** ^1^ College of Pharmacy, Dongguk University-Seoul, 04620, Seoul, South Korea; ^2^ National Cancer Center, Goyang, 10408, South Korea

**Keywords:** YDJC, CDC16, keratin reorganization, migration, invasion

## Abstract

Lung cancer is a fatal disease with a high mortality rate. The perinuclear reorganization of keratin 8 (K8) is an important biochemical phenomenon reflecting changes in the physical properties of metastatic cancer. However, there is not much of information about the regulatory molecules involved in phosphorylation and perinuclear reorganization of K8.

In this study, we investigated the role and molecular mechanisms of YdjC chitooligosaccha- ride deacetylase homolog (YDJC) in sphingosylphosphorylcholine (SPC)-induced phosphorylation and reorganization of K8, and migration and invasion (SPC-induced events). SPC induced expression of YDJC in a dose- and time-dependent manner. Gene silencing of YDJC suppressed SPC-induced events. YDJC overexpression induced the SPC-induced events. YDJC deacetylase dominant negative mutant (YDJCD13A) did not induce SPC-induced events. YDJC siRNA reduced ERK activation and overexpression of YDJC induced ERK activation. The siRNA of ERK1 or ERK2 suppressed YDJC-induced phosphorylation and reorganization of K8, and migration and invasion. Co-immunoprecipitation revealed that YDJC binds to CDC16. Interestingly, CDC16 siRNA induced SPC-induced events. Overexpression of CDC16 blocked SPC-induced events. KMPLOT analysis based on public microarray data revealed the poor prognosis of lung cancer patients with high expression of YDJC compared with patients with low expression of YDJC.

The collective results indicate that YDJC is involved in SPC-induced events in A549 lung cancer cells by interacting with CDC16. YDJC overexpression might be involved in the progression of lung cancer. These results also suggest that suppression of YDJC or boosting of CDC16 interaction with YDJC might be a novel way to prevent progression of lung cancer.

## INTRODUCTION

Lung cancer is the second most common cancer globally and accounts for 14% of new cancers [1]. Non-small cell lung cancer accounts for approximately 85% of all lung cancers, with adenocarcinomas being the most common type of lung cancer [1]. Primary lung cancers most commonly metastasize to the brain, bones, liver, and adrenal glands [2].

Metastasis is responsible for more than 90% of all cancer-related deaths [3]. Nevertheless, the processes of invasion and metastasis are still not well understood leading to hindrance in the development of new therapeutics targeting metastasis [3]. Recent research has attempted to understand the physical properties of metastatic cancer cells and to screen compounds capable of modulating the physical properties of metastasizing cancer cells [4–9].

The importance of viscoelasticity in several metastatic cancer cell lines including A549 adenocarcinoma cells is related to keratin phosphorylation and reorganization [4, 10–12]. For example, shear stress that induces reorganization of the keratin intermediate filament network requires phosphorylation of keratin 8 (K8) in A549 lung cancer cells by protein kinase C [13]. In addition, several compounds, including sphingosylphosphorylcholine (SPC) has been reported to induce perinuclear reorganization of K8 in PANC-1 human epithelial pancreatic cancer cells [4, 6, 14, 15]. The resulting changes in the mechanical deformability of cells have been examined as possible pathways that facilitate migration and increase in the metastatic competence of pancreatic tumor cells [12, 14, 16, 17].

Serine 431 (S431) of K8 is an important site in the perinuclear reorganization of K8 in PANC-1 cells [4]. Several protein kinases are involved in phosphorylation and reorganization of K8 including extracellular signal-regulated kinase (ERK) and c-Jun N-terminal kinase (JNK). Protein phosphatase 2A (PP2A) inactivates ERK and JNK by dephosphorylation [6, 18–20]. However, the complete process of K8 phosphorylation and reorganization is not completely understood.

YdjC chitooligosaccharide deacetylase homolog (YDJC) belongs to YDJC family. YDJC catalyzes the deacetylation of acetylated carbohydrates, an important step in the degradation of oligosaccharides [21]. There exists no information about the biochemical properties of YDJC enzyme. A few genetic studies have suggested that YDJC is involved in several diseases including ulcerative colitis, psoriasis, and Crohn’s disease [22, 23]. However, there are no reports on the role of YDJC in carcinogenesis and progression of cancer, especially lung cancer.

In this report, we describe SPC-induced YDJC expression in lung cancer cells. YDJC is involved in SPC-induced phosphorylation and reorganization of K8 leading to enhanced migration and invasion. CDC16 binds to YDJC to inhibit actions of YDJC. Gene silencing of CDC16 leads to phosphorylation and reorganization of K8 via ERK activation. YDJC overexpression might be involved in the progression of lung cancer.

## RESULTS

### SPC induces YDJC expression in lung cancer cells

**Figure 1:**
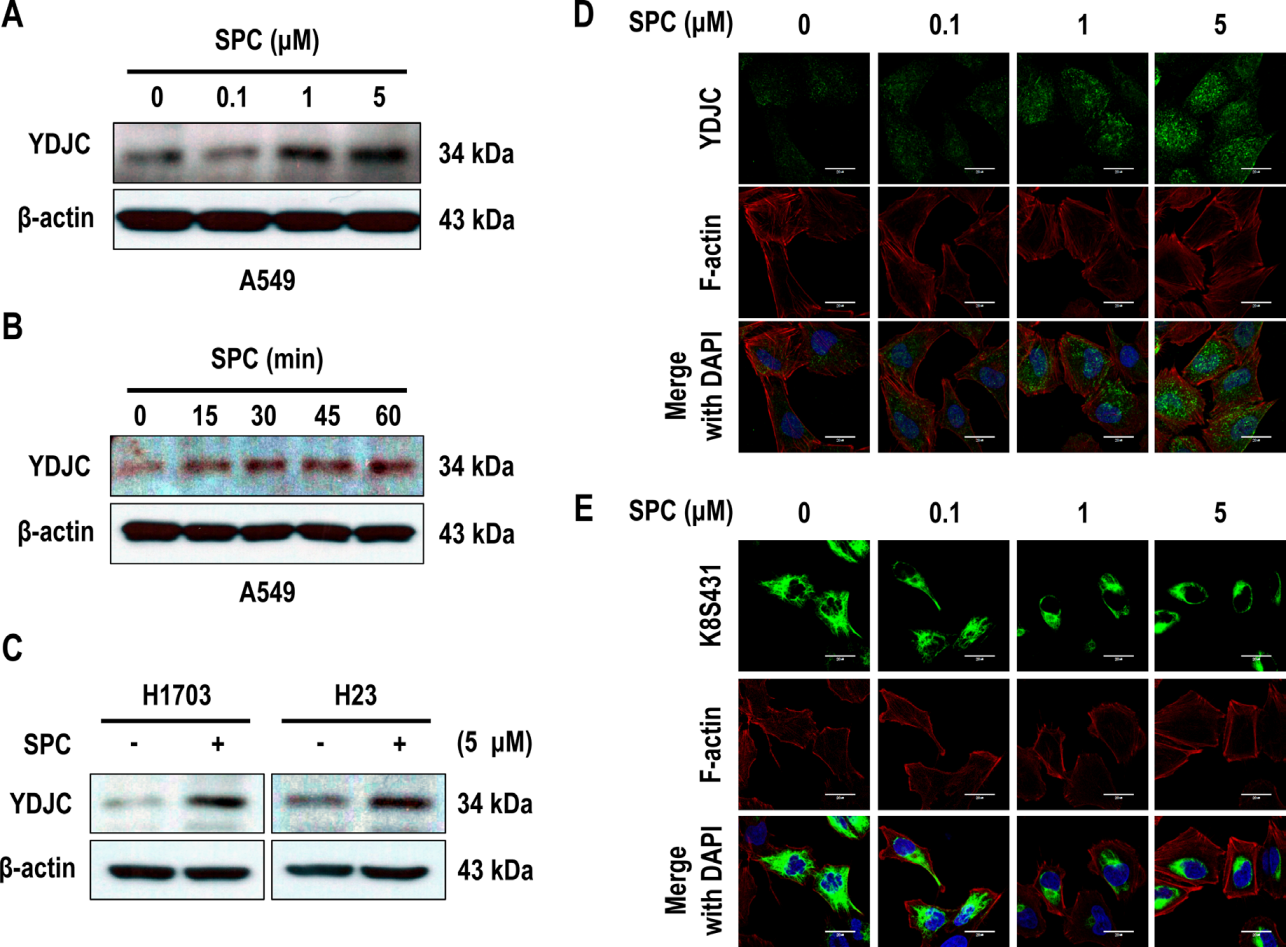
SPC induces YDJC expression in lung cancer cells (**A**) Dose-dependent expression of YDJC protein in A549 cells stimulated with the indicated concentrations of SPC for 1 h. (**B**) Time-dependent expression of YDJC protein in A549 cells treated with 5 μM SPC for the times indicated. (**C**) Effects of SPC on the expression of YDJC protein in H1703 and H23 cells. (**D**) Confocal microscopic analysis of YDJC expression in A549 cells stimulated with the indicated concentration of SPC for 1 h. Nuclei were stained with DAPI (blue). (**E**) Confocal microscopic analysis of perinuclear keratin reorganization in A549 cells stimulated with the indicated concentration of SPC for 1 h. A549 cells were stained with an anti-K8S431 antibody coupled with FITC-conjugated anti-Rabbit IgG (green), and DAPI (blue). F-actin was stained with Texas Red-X phalloidin. Scale bars, 10 μm. In (A) and (C), YDJC expression was detected by Western blotting analysis.

Microarray experiments demonstrated increased YDJC expression in cells treated with SPC (data not shown). SPC induced the expression of YDJC in A549 cells in a dose- and time-dependent manner (Figure 1A, 1B). The induction of YDJC expression was also observed in H1703 and H23 lung cancer cells (Figure 1C). YDJC expression was examined by confocal microscopy. A549 cells showed the typical pan-cytoplasmic pattern of K8 filaments and weak YDJC expression (Figure 1D, 1E). SPC induced YDJC expression and the reorganization of K8 filaments to a perinuclear, ring-like structure (Figure 1D, 1E).

### YDJC is involved in SPC-induced events of lung cancer cells

**Figure 2:**
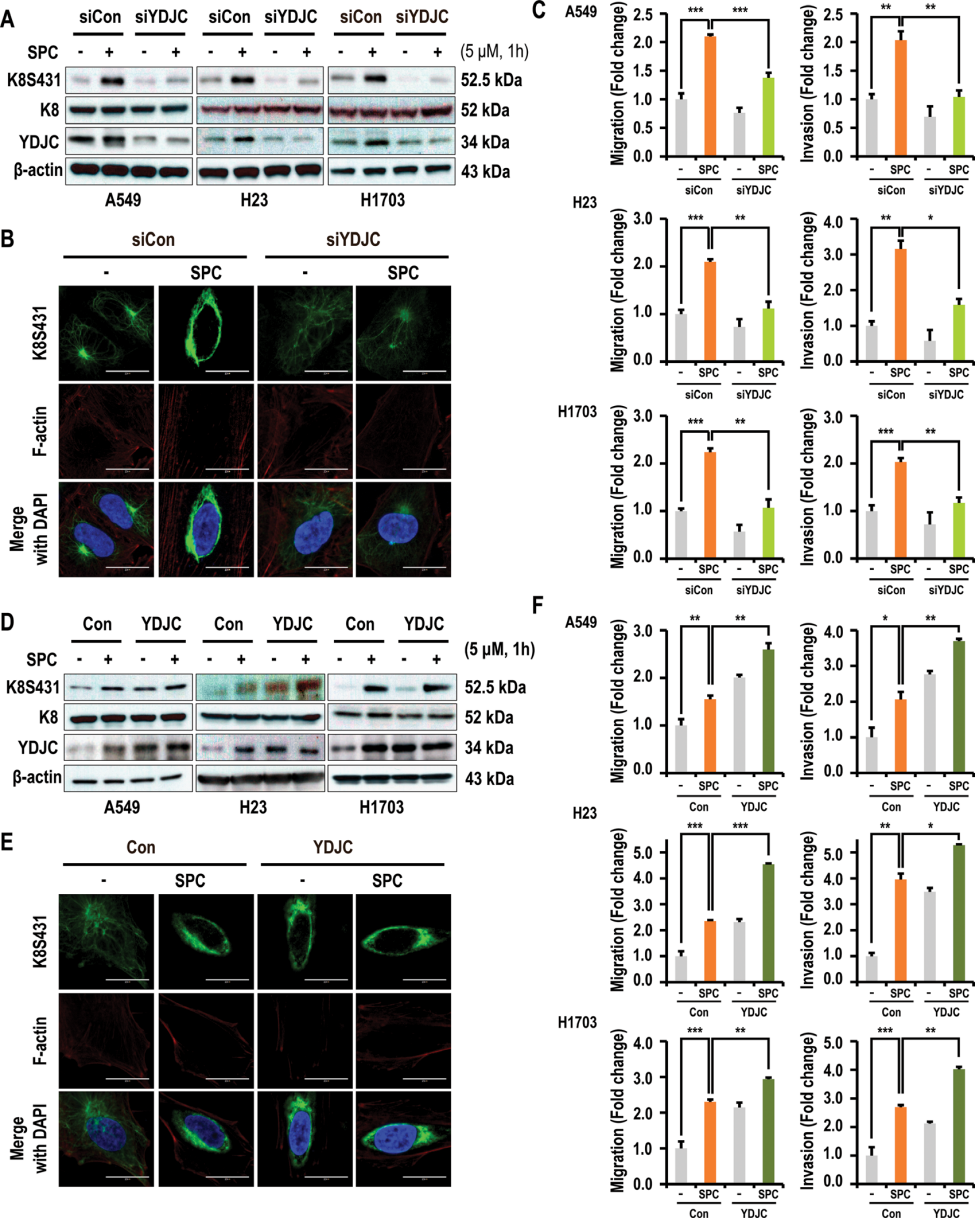
YDJC is involved in the SPC-induced K8 phosphorylation and reorganization of A549 cells (**A**) Effect of YDJC siRNA on SPC-induced K8 phosphorylation. For gene silencing of YDJC, the A549, H1703, and H23 cells were transfected with the indicated amount of YDJC siRNA or control siRNA and stimulated with or without 5 μM SPC for 1 h. (**B**) Effect of YDJC siRNA on perinuclear keratin organization in A549 cells stimulated with SPC. A549 cells were stained with anti-K8S431 antibody coupled with FITC-conjugated anti-Rabbit IgG (green) and DAPI (blue). F-actin was stained with Texas Red-X phalloidin. (**C**) Effects of YDJC siRNA on SPC-induced migration and invasion in A549 cells. (**D**) Effect of YDJC overexpression on SPC-induced K8 phosphorylation. For gene overexpression of YDJC, the A549, H1703, and H23 cells were transfected with the plasmid containing YDJC and control empty vector (4 mg) and subsequently treated with SPC (5 μM) for 1 h. (**E**) Effect of YDJC overexpression on perinuclear keratin organization in A549 cells stimulated with SPC. A549 cells were stained with an anti-K8S431 antibody coupled with FITC-conjugated anti-Rabbit IgG (green) and DAPI (blue). (**F**) Effects of YDJC overexpression on SPC-induced migration and invasion in A549 cells. After overexpression of YDJC and subsequent to treatment with SPC, the A549, H23, and H1703 cells were plated in the upper chamber of Transwell insert for migration and invasion assay. In (B) and (E), A549 cells were stained with the indicated antibodies. Nuclei were stained with DAPI (blue). Scale bars, 10 μm. In (C) and (F), cells were subsequently counted under four randomly chosen high-power fields (20X). The results are representative of three independent experiments with similar results. ^#^*P* < 0.05 compared with the control group. ^*^P < 0.05 compared with the SPC-treated group.

To investigate the involvement of YDJC in SPC-induced events including phosphorylation and reorganization of K8, and migration and invasion, the effect of YDJC gene silencing and overexpression on the SPC-induced events were examined in lung cancer cells. Gene silencing of YDJC inhibited SPC-induced phosphorylation of K8 in A549, H23, and H1703 lung cancer cells (Figure 2A), and reorganization of K8 in A549 lung cancer cells (Figure 2B, Supplementary Figure 1 in Supplementary Information). In addition, SPC-induced migration and invasion were suppressed by gene silencing of YDJC in A549 cells (Figure 2C). In contrast, overexpression of YDJC induced phosphorylation in A549, H23, and H1703 lung cancer cells (Figure 2D) and reorganization of K8 in A549 cells were observed even without SPC treatment (Figure 2E, Supplementary Figure 1 in Supplementary Information). Overexpression of YDJC promoted migration and invasion in A549 cells (Figure 2F). These results implicate the involvement of YDJC in the SPC-induced events.

### Deacetylase function of YDJC is essential to induce phosphorylation and reorganization of K8

**Figure 3:**
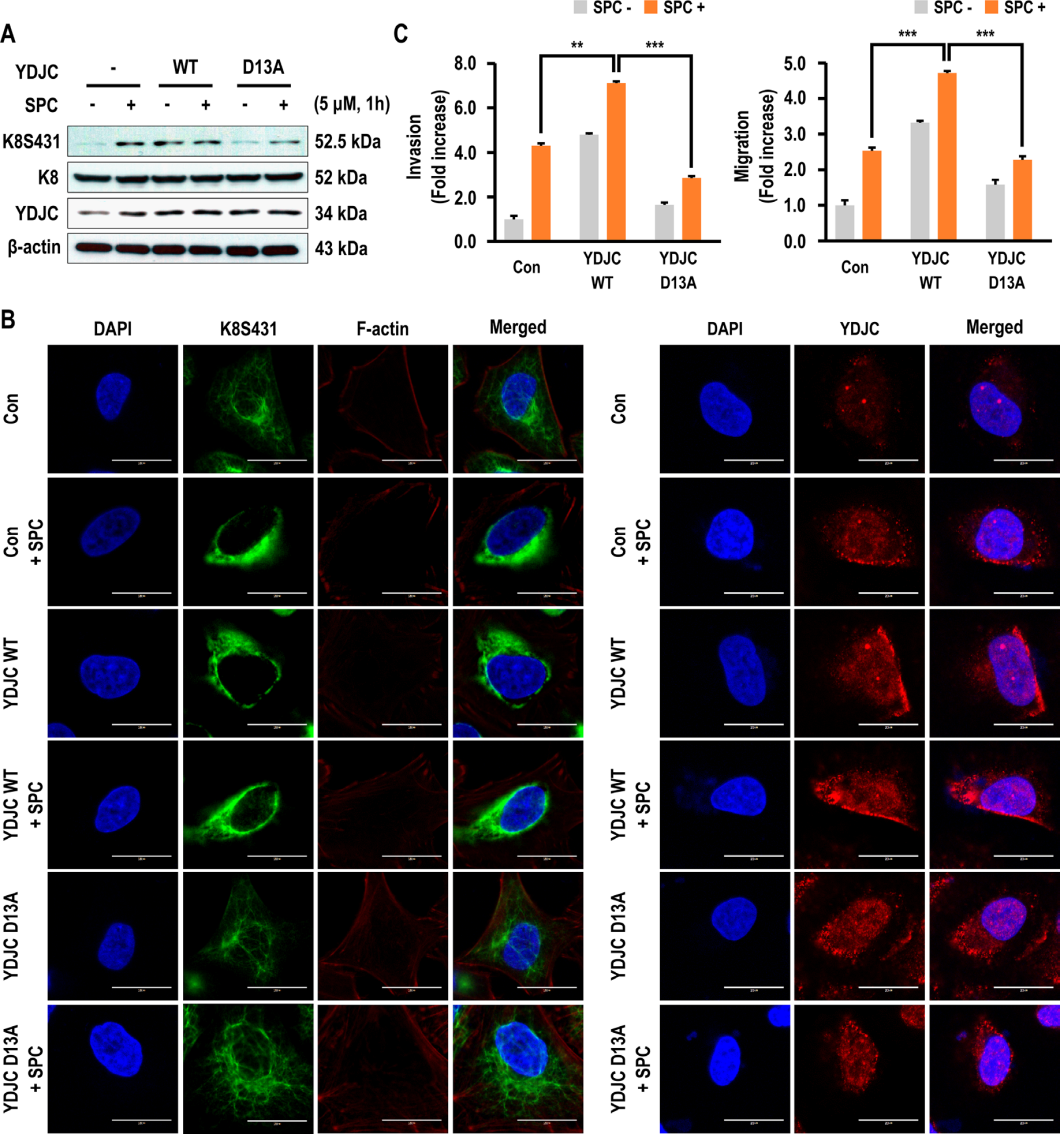
Effects of deacetylase activity of YDJC on phosphorylation and reorganization of K8 (**A**) Effect of deacetylase activity of YDJC on SPC-induced K8 phosphorylation. Cell lysates were analyzed by Western blotting. (**B**) Effect of deacetylase activity of YDJC on SPC-induced K8 reorganization. A549 cells were stained with the indicated antibodies. Nuclei were stained with DAPI (blue). Scale bars, 10 μm. (**C**) Effect of deacetylase activity of YDJC on SPC-induced migration and invasion. The results are representative of three independent experiments with similar results. ^#^*P* < 0.05 compared with the control group. ^*^P < 0.05 compared with the SPC-treated group. In A and B, for gene overexpression of YDJC, and YDJCD13A (a dominant negative form of deacetylase), A549 cells were transfected with the plasmid containing HA-tagged YDJC or YDJC_D13A_ and control empty vector (4 mg) and subsequently treated with SPC (5 μM) for 1 h.

YDJC belongs to the YDJC superfamily and exerts deacetylase activity [21, 24]. Therefore, we examined whether deacetylase activity of YDJC could enhance SPC-induced K8 phosphorylation. Aspartic acid (D) 13 of YDJC was site-directed mutated to alanine YDJC_D13A_ as the dominant negative form [25]. K8 phosphorylation was enhanced by overexpression of YDJC even without SPC treatment, but overexpression of YDJC_D13A_ could not induce K8 phosphorylation (Figure 3A). Also, SPC-induced K8 reorganization was suppressed by YDJC_D13A_ in A549 cells (Figure 3B, Supplementary Figure 2 in Supplementary Information). YDJC_D13A_ overexpression suppressed SPC-induced migration and invasion of A549 lung cancer cells (Figure 3C). These results suggest that deacetylase activity of YDJC is involved in SPC-induced phosphorylation and reorganization of K8 leading to migration and invasion of A549 cells.

### ERK pathway is involved in YDJC-induced phosphorylation and reorganization of K8

**Figure 4:**
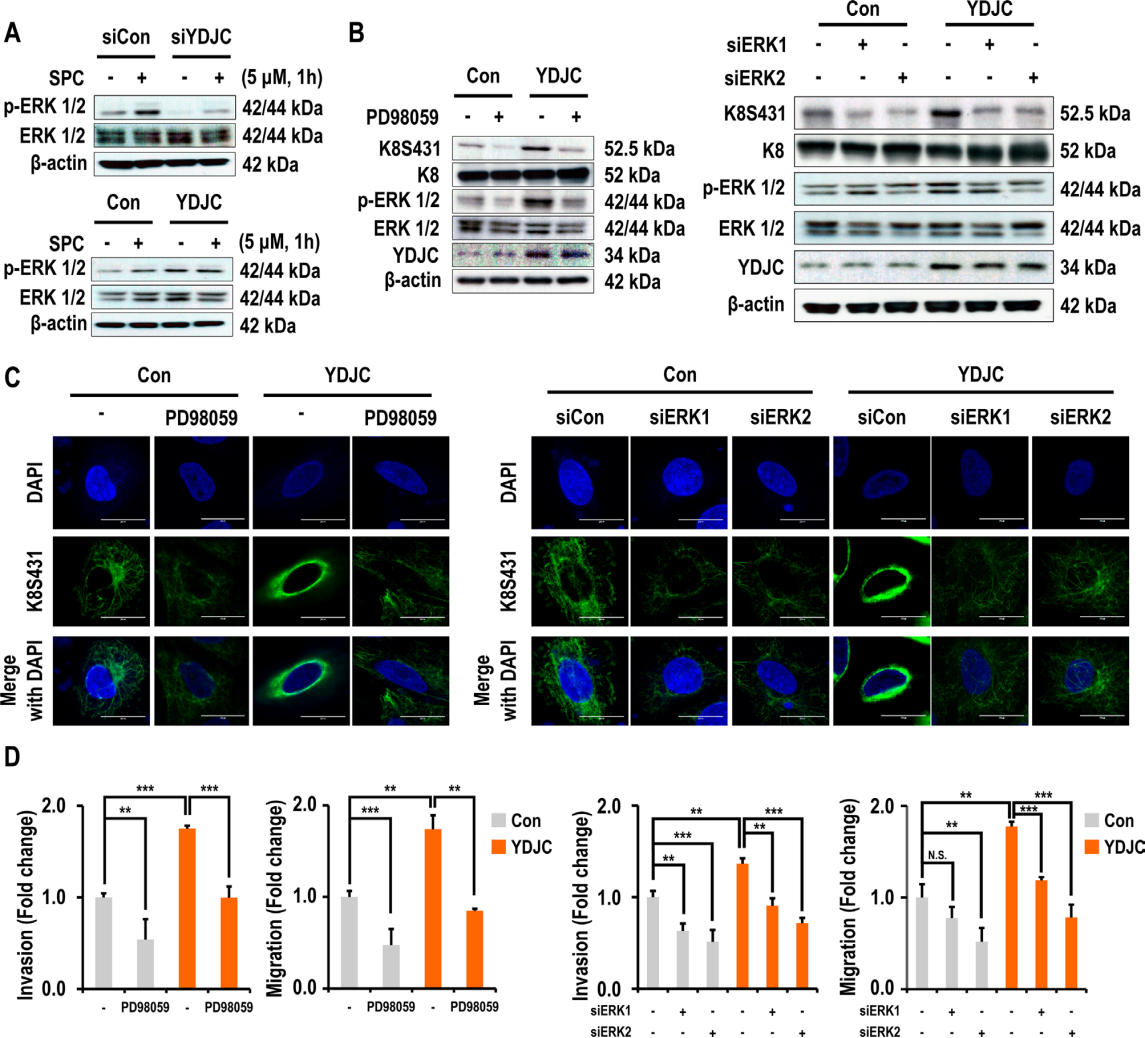
Effects of deacetylase activity of YDJC on phosphorylation and reorganization of K8 (**A**) Effect of YDJC on SPC-induced ERK1/2 phosphorylation. For gene silencing of YDJC, A549 cells were transfected with YDJC siRNA and control siRNA and subsequently treated with SPC for 1 h. For gene overexpression of YDJC, A549 cells were transfected with a plasmid containing YDJC and control empty vector and then treated with SPC for 1 h. (**B**) Effect of PD98059 and siRNA of ERK1 or ERK2 on YDJC induced-K8 phosphorylation. A549 cells were transfected with a plasmid containing YDJC and control empty vector and then treated with PD98059 (10 μM), or ERK1 and ERK2 siRNA. (**C**) Effect of PD98059 and siRNA of ERK1 or ERK2 on YDJC-induced K8 perinuclear keratin organization. A549 cells were stained with an anti-K8S431 antibody coupled with FITC-conjugated anti-Rabbit IgG (green) and DAPI (blue). (**D**) Effect of PD98059 and siRNA of ERK1 or ERK2 on YDJC-induced invasion and migration. A549 cells were plated in the upper chamber of Transwell insert for migration and invasion assay. The results shown are representative of three independent experiments with similar results (*n* = 3).

Activation of ERK and JNK are involved in SPC-induced keratin phosphorylation and reconstruction [4, 6, 12]. Therefore, we investigated the effect of YDJC expression on the activation of ERK and JNK and expression of PP2A. The initial experiment examined whether ERK and JNK signaling is involved in keratin phosphorylation by SPC using inhibitors of mitogen-activated protein kinase (MAPK). PD98059, a MEK inhibitor which blocks the phosphorylation of ERK and SP600125, JNK inhibitors suppressed SPC-induced K8 phosphorylation in A549 lung cancer cells (Supplementary Figure 3 in Supplementary Information). In particular, marked inhibition of ERK was observed. Subsequent studies focused on the effect of YDJC on ERK. Gene silencing of YDJC suppressed the expression of phosphorylated ERK, an activated form of ERK and overexpression of YDJC induced activation of ERK (Figure 4A). Interestingly, the increased expression of YDJC caused activation of ERK and phosphorylation of K8 even without treatment with SPC. Thus we examined whether ERK inhibitor or siRNA of ERK suppressed YDJC-induced phosphorylation and reorganization of K8. ERK inhibitor and ERK siRNA inhibited phosphorylation and reorganization of K8 induced by YDJC (Figure 4B, 4C). ERK inhibition by ERK inhibitor and ERK siRNA also suppressed the YDJC-induced migration and invasion (Figure 4D). These results suggest that the ERK pathway is involved in the YDJC-induced phosphorylation and reorganization of K8 in lung cancer cells.

### YDJC binds to CDC16

**Figure 5:**
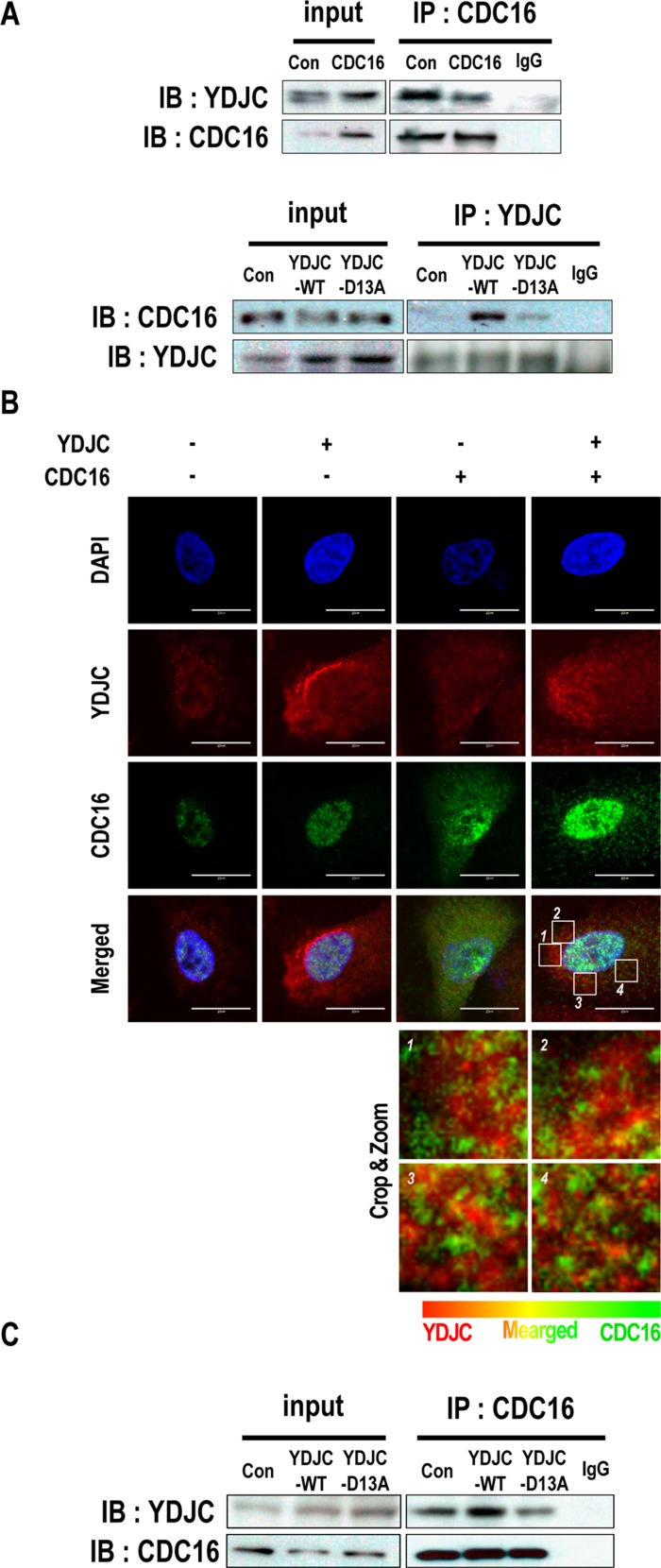
YDJC binds to CDC16 (**A**) Binding of YDJC to CDC16. Immunoprecipitation with the CDC16 antibody (IP: CDC16) or YDJC antibody (IP: YDJC) was performed on the extracts of A549 cells transfected with plasmids containing CDC16, or wild-type YDJC. The resulting immunocomplexes were immunoblotted with YDJC, or CDC16 antibodies, respectively. (**B**) Localization of YDJC and CDC16. A549 cells were stained with an anti-YDJC antibody (red) and anti-CDC16 antibody (green) coupled with FITC-conjugated anti-Rabbit IgG, FITC-conjugated anti-Mouse IgG, and DAPI (blue). (**C**) Effects of deacetylase activity of YDJC subsequent to binding to CDC16. Immunoprecipitation with the CDC16 antibody (IP: CDC16) was performed on the extracts of A549 cells transfected with plasmids containing wild-type YDJC or YDJC_D13A_. The resulting immunocomplexes were immunoblotted with YDJC and CDC16 antibodies. Effect of overexpression of wild-type YDJC or YDJC_D13A_ on the expression of CDC16. For gene overexpression of YDJC and YDJC_D13A_ (dominant negative), A549 cells stably expressing CDC16 were transfected with a plasmid containing YDJC or YDJC_D13A_ and control empty vector and subsequently treated with SPC for 1 h. Localization of YDJC and CDC16. A549 cells were stained with an anti-YDJC antibody (red) and anti-CDC16 antibody (green) coupled with FITC-conjugated anti-Rabbit IgG, FITC-conjugated anti-Mouse IgG, and DAPI (blue). (Bottom; In the cytoplasm, the merged site was zoomed and show in detail).

To elucidate the involvement of YDJC in the SPC-induced events, we first tried to determine the proteins that binds with YDJC. Huttlin et al. reported that CDC16 binds to YDJC, so we confirmed the result by co-immunoprecipitation (co-IP) [26]. To confirm the binding, co-IP was performed using CDC16 antibody and YDJC antibody. Co-IP of YDJC with CDC16 was carried out without SPC treatment (Figure 5A). The interactions between YDJC and CDC16 were confirmed by confocal microscopy (Figure 5B). Although CDC16 was strongly expressed in the nucleus, it was also expressed in the cytosol, and YDJC expression was merged with CDC16 in the cytosol as well as in the nucleus (Figure 5B).

Subsequently, we examined the effects of the deacetylase activity of YDJC on its binding to CDC16 using YDJC_D13A_. The binding of YDJC to CDC16 was affected by its deacetylase activity since YDJC_D13A_ showed decreased binding to CDC16 (Figure 5C).

### Involvement of CDC16 in SPC or YDJC-induced phosphorylation and reorganization of K8, migration, and invasion

**Figure 6:**
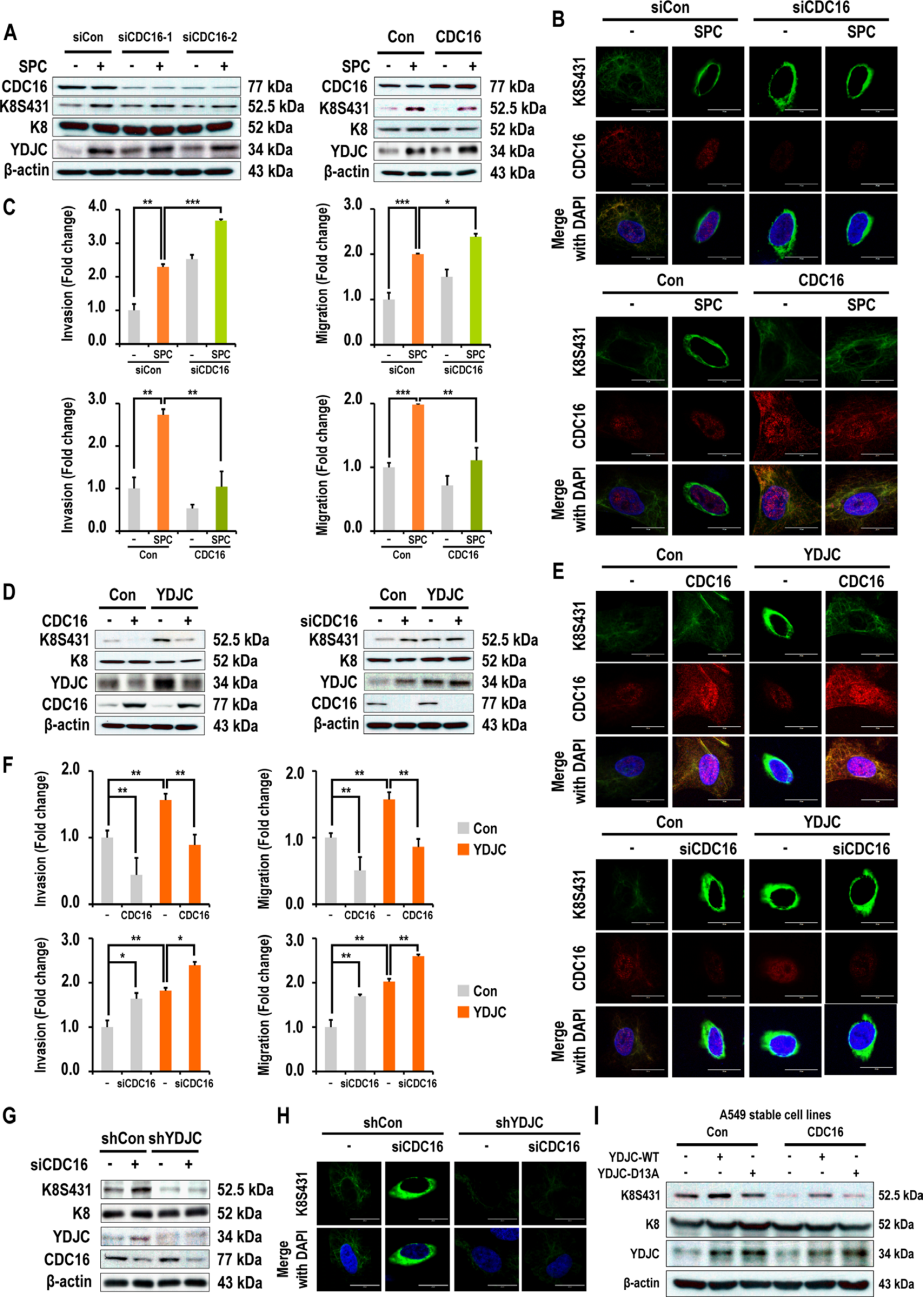
Involvement of CDC16 in SPC or YDJC-induced phosphorylation and reorganization of K8, and migration and invasion (**A**) Effects of CDC16 on SPC-induced K8 phosphorylation in A549 lung cancer cells. (**B**) Effects of CDC16 on SPC-induced K8 reorganization in A549 lung cancer cells. (**C**) Effects of CDC16 on SPC-induced migration and invasion of A549 lung cancer cells. (**D**) Effects of CDC16 overexpression on YDJC-induced K8 phosphorylation of A549 lung cancer cells. (**E**) Effects of CDC16 on YDJC-induced K8 reorganization of A549 lung cancer cells. (**F**) Effects of CDC16 on YDJC-induced migration and invasion of A549 lung cancer cells. (**G**) Effects of CDC16 silencing on K8 phosphorylation of YDJC-silenced A549 lung cancer cells. (**H**) Effects of CDC16 silencing on K8 reorganization of YDJC-silenced A549 lung cancer cells. (**I**) Effects of YDJC overexpression on K8 phosphorylation of CDC16-overexpressed A 549 lung cancer cells.

Subsequently, we examined the involvement of CDC16 expression in SPC-induced phosphorylation and reorganization of K8. Interestingly, gene silencing of CDC16 induced phosphorylation and reorganization of K8, migration, and invasion (Figure 6A, 6B, 6C, Supplementary Figure 4 in Supplementary Information). Next, we investigated whether CDC16 is involved in YDJC-induced phosphorylation and reorganization of K8, and migration and invasion of A549 cells (Figure 6D). siRNA of CDC16 induced phosphorylation and reorganization of K8, migration, and invasion of A549 lung cancer cells. Furthermore, overexpression of CDC16 inhibited YDJC-induced events (Figure 6D, 6E, 6F, Supplementary Figure 4 in Supplementary Information).

In order to clarify the relationship between YDJC and CDC16, we examined whether reduced expression of YDJC affects the CDC16 siRNA-induced phosphorylation of K8. Reduced expression of YDJC inhibited CDC16 siRNA-induced phosphorylation and reorganization of K8 (Figure 6G, 6H). In contrast, CDC16 overexpression suppressed YDJC_WT_-induced K8 phosphorylation (Figure 6I).

### Effects of expression of YDJC on the prognosis of lung cancer patients

To investigate the prognostic significance of YDJC mRNA expression in lung cancer, survival analysis was done using online Kaplan Meier-plotter [27]. Lung cancer patients were divided into two subgroups (YDJC-high and YDJC-low) based on their median expression level.

**Figure 7:**
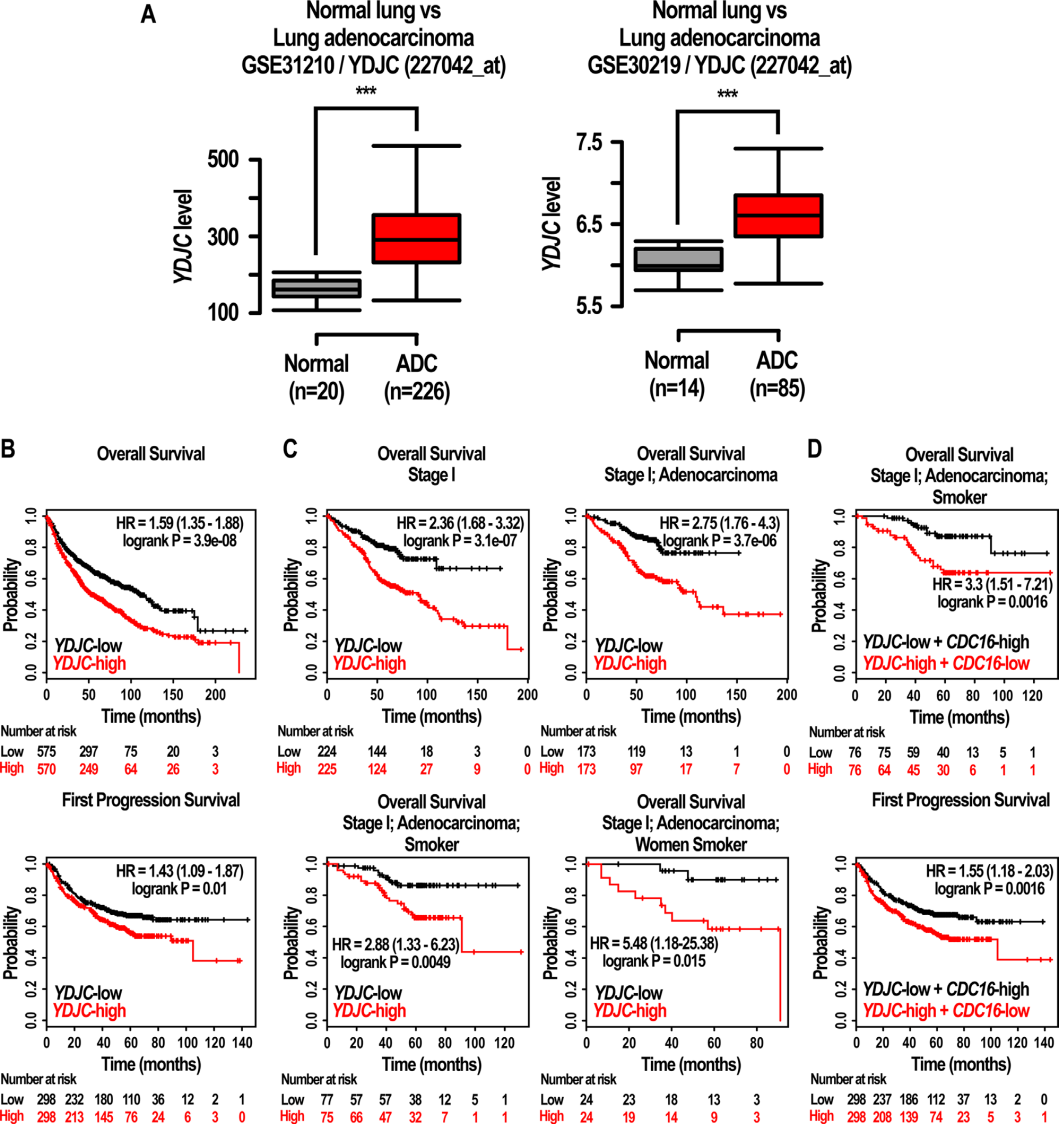
Effects of expression of YDJC on the prognosis of lung cancer patients (**A**) Gene expression of YDJC (227042_at) in normal lung tissue and lung adenocarcinoma. The box plot shows the expression values of YDJC in normal lung or lung adenocarcinoma tissues from two independent datasets (GSE31210; YDJC (227042_at) and GSE30219; YDJC (227042_at)). Normal: normal lung tissue; ADC: lung adenocarcinoma (**B**) Effects of YDJC on overall survival, and first progression of lung cancer patients. The prognostic value of YDJC was estimated in a large public clinical microarray database of lung cancers from 1125, 596 patients, respectively (**C**) Effects of YDJC expression (227042_at) on overall survival of restricted subtypes in lung cancer patients: ‘stage 1’ (*n* = 449, HR = 2.39, *p* = 0), ‘stage 1 and adenocarcinoma’ (*n* = 346, HR = 2.75, *p* = 0), ‘stage 1, adenocarcinoma, and smokers’ (*n* = 2.88, HR = 2.88, *p* = 0.0049), ‘stage 1, adenocarcinoma, and women smokers (*n* = 48, HR = 5.48, *p* = 0.015) (**D**) Effects of YDJC/CDC16 expression (227042_at/209659_s_at) on overall survival (OS) and first progression (FP) of restricted subtypes in lung cancer patients: ‘stage 1, adenocarcinoma, and smokers’ (*n* = 152, HR = 3.3, *p* = 0.0016 for OS), (*n* = 596, HR = 1.55, *p* = 0.0016).

Initially, we explored whether YDJC expression is related to lung cancer. Interestingly, YDJC expression was increased in patients lung adenocarcinoma (GEO Datasets: GSE31210; YDJC probe ID: 227042_at, GSE30219; YDJC probe ID: 227042_at, shown in Figure 7A). We also found that the overall survival was high in lung cancer patients having low expression level of YDJC (*n* = 1145, *p* = 0, HR = 1.59 (1.35–1.88)). Progression free survival was decreased in patients having high expression level of YDJC (*n* = 596, *p* = 0.01, HR = 1.43 (1.09–1.87)) (Figure 7B). Stage 1 patients with lung cancer having high YDJC expression (*n* = 449, *p* = 0, HR = 2.36 (1.68–3.32)) and patients with stage 1 adenocarcinoma (*n* = 346, *p* = 0, HR = 2.75 (1.76 to 4.3)) showed higher HR compared to all other patients (*n* = 1145, *p* = 0, HR = 1.59 (1.35–1.88)) (Figure 7C). Patients with stage 1 adenocarcinoma (*n* = 152, *p* = 0.0049, HR = 2.88 (1.33 to 6.23)) and female smokers (*n* = 48, *p* = 0.015, HR = 5.48 (1.18–25.38)) also showed higher HR compared to all other patients (*n* = 1145, *p* = 0, HR = 1.59 (1.35–1.88)). However, in latter case, the number of n was insufficient and further research is necessitated in the future.

In addition, we compared ‘YDJC-high and CDC16-low’ patients group with ‘YDJC-low and CDC16-high’ patients group using KMPLOT. Lung cancer patients with YDJC-high and CDC16-low stage 1, adenocarcinoma, and history of smoking showed higher HR value and improved *p-*value in overall survival compared to lung cancer patients with YDJC-high, stage 1, adenocarcinoma, and history of smoking (increase from 2.88 to 3.3 and decrease from 0.0049 to 0.0016) (Figure 7D). Furthermore, progression-free survival in lung cancer patients with ‘YDJC-high and CDC16-low’ showed higher HR value and improved *p*-value compared to lung cancer patients with YDJC-high (increase from 1.43 to 1.5 and decrease from 0.01 to 0.0016) (Figure 7D).

## DISCUSSION

Viscoelasticity of metastatic cancer cells, such as PANC-1 cells, is regulated by SPC. The physical properties of several metastatic cancer cells are related to keratin phosphorylation and perinuclear reorganization [4, 6]. The molecular mechanisms involved in keratin phosphorylation and perinuclear reorganization are largely unknown although some kinases including ERK, JNK, and protein kinase C (PKC) are known to be involved in these events [6, 12, 14, 16].

We have been interested in finding new players involved in keratin phosphorylation and perinuclear reorganization, since these phenomena are correlated with viscoelasticity of metastatic cancer cells and identification of novel players involved in keratin reorganization might be a new target of controlling the metastasis of cancer cells [4, 8]. Previously, we have shown that FTY720 can change Young’s modulus coefficient in a PANC-1 cell by suppressing SPC-induced phosphorylation and reorganization of K8 via G protein coupled receptor 12 [8].

In the present study, an increase in YDJC in A549 or other lung cancer cells with SPC treatment was observed (Figure 1A). Gene silencing of YDJC suppressed the SPC-induced phosphorylation and reorganization of K8 and ectopic expression of YDJC-induced and SPC-induced phosphorylation and reorganization of K8 (Figure 2A, 2B). Furthermore, gene silencing of YDJC inhibited the SPC-induced migration and invasion and overexpression of YDJC-increased migration and invasion, even without SPC treatment (Figure 2C, 2F). Overall, the results suggest that YDJC is involved in SPC-induced phosphorylation and reorganization of K8 in A549 cancer cells.

We examined the effects of deacetylase activity of YDJC on SPC-induced K8 phosphorylation and reorganization since YDJC belongs to the YDJC family that catalyzes the deacetylation of acetylated carbohydrates, which is an important step in the degradation of oligosaccharides [21]. It was observed that the deacetylase function of YDJC is important in SPC-induced K8 phosphorylation and reorganization, migration and invasion (Figure 3). To the best of our knowledge, there are no reports regarding the relationship between the deacetylase activity of YDJC and keratin-related events. Therefore, our study represents the first report regarding the involvement of the deacetylase activity of YDJC in keratin reorganization.

For the question that how is YDJC involved in keratin phosphorylation and reorganization? We speculate the possible interaction of YDJC with molecules affecting kinase pathways, such as ERK and JNK with subsequent phosphorylation of K8. Furthermore, CDC16 has been proposed as a binding partner of YDJC in breast cancer cells [26]. We confirmed the binding of YDJC to CDC16 by co-IP (Figure 5A). Deacetylase activity influences the binding of YDJC to CDC16 which is involved in SPC-induced events (Figure 5B).

It is interesting to note that YDJC binds directly to CDC16 (Figure 5A). CDC16 might activate PP2A leading to dephosphorylation of K8 via inactivation of ERK and JNK or directly [8, 14, 28]. Our data suggest that YDJC might block the CDC16’s action via suppressing ERK and JNK with subsequent phosphorylation and reorganization of K8 (Figure 6A, 6B).

**Figure 8:**
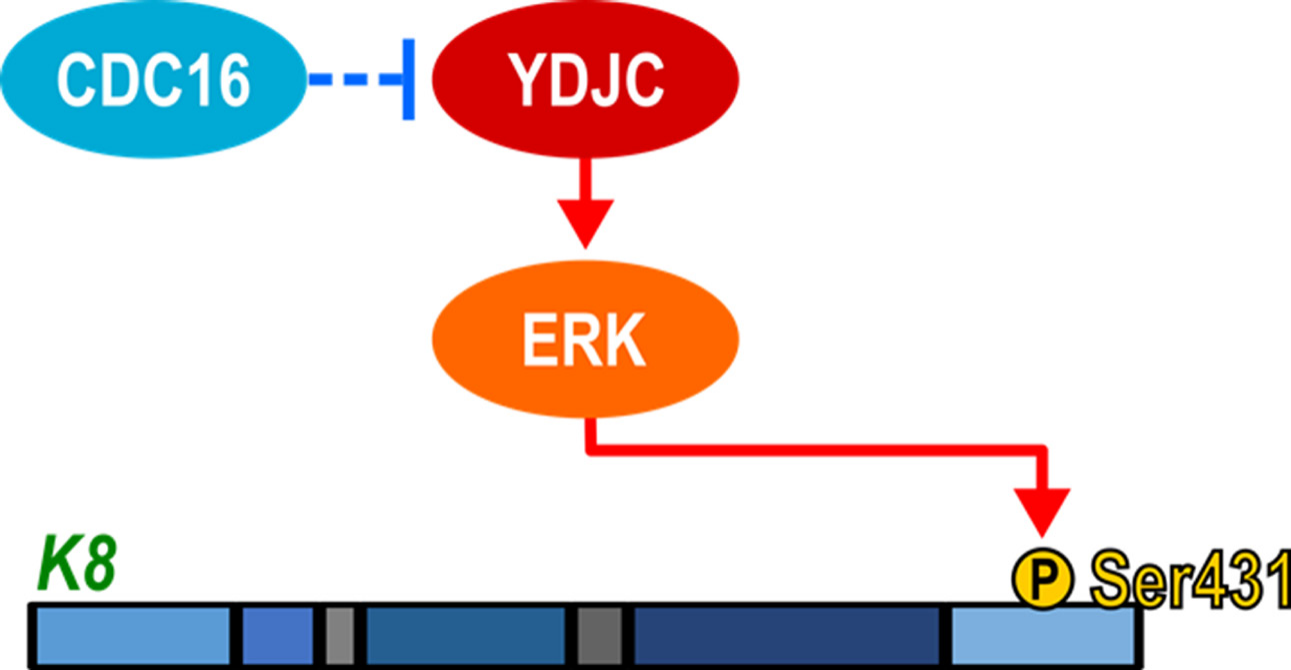
Schematic diagram of involvement of YDJC in SPC-induced K8 phosphorylation

We examined the involvement of CDC16 in SPC-induced K8 phosphorylation and reorganization since CDC16 binds to YDJC (Figure 5A). CDC16 siRNA induced phosphorylation and reorganization of K8. CDC16 overexpression suppressed the SPC-induced events (Figure 6A). It is not clear as for how CDC16 suppresses the phosphorylation of S431 in K8. To clarify the relationship between CDC16 and YDJC, we examined whether overexpression of CDC16 inhibited YDJC-induced K8 phosphorylation. Overexpression of CDC16 inhibited YDJC-induced phosphorylation and reorganization of K8 (Figure 6D, 6E). CDC16 siRNA-induced K8 phosphorylation was inhibited by YDJC gene silencing (Figure 6G). These results suggested that YDJC is involved in CDC16 siRNA-induced K8 phosphorylation. Next, we examined whether overexpression of CDC16 inhibits YDJC-induced K8 phosphorylation. As expected, overexpression of CDC16 inhibited the phosphorylation of K8 induced by YDJC (Figure 6D, 6I). These results suggested that CDC16 might be located in the upstream of YDJC and inhibit the action of YDJC on K8 (Figure 8).

Another interesting possibility of YDJC is the direct modification of K8 by YDJC. The nature of substrates of YDJC is not yet clear, although YDJC is known as carbohydrate deacetylase. K8 undergoes O-GlcNAc (N-acetylglucosamine) modification [29, 30]. YDJC is a chitooligosaccharide deacetylase homolog. Chitooligosaccharide deacetylase hydrolyzes the N-acetamido groups on the nascent chitin chains [31]. It remains unclear if K8 will serve as a substrate for YDJC. For K8 to act as a substrate for YDJC, it must bind to YDJC and possess O-GlcNAc in K8 although not chitin. Interestingly, K8 has an O-GlcNAc residue. Therefore, it is necessary to study whether the N-acetyl moiety of O-GlcNAc of K8 is deacetylated by YDJC.

The observation that lung cancer patients with YDJC high expression showed poor prognosis compared with patients with YDJC low expression group suggested that YDJC may be a good target for the development of anticancer drug for lung cancer (Figure 7).

The collective findings indicate that YDJC is involved in SPC-induced events in A549 lung cancer cells by binding to CDC16. These results suggest that suppression of YDJC or boosting of CDC16 interaction with YDJC might be a novel strategy to prevent the malignant changes in the physical properties of metastatic cancer cells (Figure 8).

## MATERIALS AND METHODS

### Chemical and reagents

RPMI-1640 medium, fetal bovine serum (FBS), phosphate-buffered saline (PBS), and antibiotics (penicillin and streptomycin) were purchased from Welgene Inc. (Seoul, Korea). Lipofectamine™ 2000 was purchased from Invitrogen (Carlsbad, CA, USA). JetPEI was purchased from Polyplus-transfection (Illkirch-Graffenstaden, France). SPC was purchased from Matreya (Pleasant Gap, PA, USA). PD98059, SP600125, and SB203580 were purchased from Calbiochem (La Jolla, CA, USA). Anti-YDJC, anti-K8, and the phosphospecific antibody to detect K8S431 were obtained from Abcam (Cambridge, MA, USA). Anti-ERK and the phosphospecific antibody to detect ERK1/2 antibodies were purchased from Cell Signaling Technology (Beverly, MA, USA). Anti-CDC16 and anti-β-actin antibody were obtained from Santa Cruz Biotechnology, Inc. (Santa Cruz, CA, USA). Alexa Fluor 488 goat anti-rabbit antibody and Alexa Fluor 594 goat anti-mouse antibody were obtained from Molecular Probes, Inc. (Eugene, OR, USA). All the chemicals were freshly prepared at the time of each experiment.

### Cell culture

The A549, H23, and H1703 human lung cell lines were obtained from the American Type Culture Collection (Manassa, VA, USA). The cells were grown and maintained at 37°C in an atmosphere of 95% air and 5% CO_2_ in RPMI-1640 supplemented with 10% heat-inactivated FBS, 100 U/ml penicillin, and 100 mg/ml streptomycin.

### Cell invasion assay

The invasion assay was performed using transwell inserts (Neuro Probe, Inc., Gaithersburg, MD, USA) coated with Matrigel (0.5 mg/mL), as described previously [30]. Cells (1 × 10^6^ cells/mL) suspended in serum-free medium were added to the upper chamber of each insert. Medium containing 10% FBS was added to the lower chamber. After 16 h incubation, cells on the upper surface of the membrane that had not migrated were scraped off with cotton swabs. Cells that had migrated to the lower surface were stained using the Hema 3 staining system (Fisher Scientific, Houston, TX, USA), photographed, and the cells in four randomly selected fields were enumerated at 20X magnification. All the experiments were repeated at least three times with at least two replicates each time.

### Cell migration assay

Migration assays were performed in transwell inserts coated with 10 μg/mL fibronectin. Cells (1 × 10^6^ cells/mL) suspended in serum-free medium were added to the upper chamber of each insert as described previously [32]. The lower chamber received a medium containing 3% FBS. After 6 h incubation, A549 cells on the upper surface of the membrane that had not migrated were scraped off. Cells that had migrated to the lower surface were stained by Diff-quick and cells in four randomly chosen fields were enumerated at 20× magnification. All the experiments were repeated at least three times with at least two replicates each time.

### Western blot analysis

Western blot analysis was performed, as described previously [33]. After incubation, the cells were washed twice with ice-cold PBS and disrupted in RIPA buffer with protease inhibitor cocktail and Xpert phosphatase inhibitor cocktail solution (GenDEPOT, Inc., Austin, TX, USA) on ice for 30 min. Cell lysates were centrifuged at 15,000 rpm for 15 min at 4°C. The supernatants were used for Western blotting. The protein concentration was measured using the Bradford method. Aliquots of the lysates (20–30 μg of protein) were separated by 8–12% sodium dodecyl sulfate-polyacrylamide gel electrophoresis (SDS-PAGE) and transferred to a polyvinylidene fluoride (PVDF) membrane (Invitrogen) with glycine transfer buffer containing 192 mM glycine, 25 mM Tris-HCl (pH 8.8), and 10% v/v methanol. After blocking the non-specific sites with 5% non-fat dry milk, the membrane was incubated with specific primary antibody in 3% bovine serum albumin (BSA) at 4°C overnight, and further incubated for 60 min with a peroxidase-conjugated secondary antibody (1:5,000, Santa Cruz Biotechnology Inc.) at room temperature. Immunoreactive proteins were detected using PowerOpti-ECL Western blotting detection reagent (Animal Genetics Inc., Gyeonggi, Korea).

### Gene silencing by small-interfering RNA (siRNA)

YDJC-1 siRNA (sequence (I): 5′- GCUUCUUCCUUGGCAAGAU-3′, (II):5′- AUCUUGC- CAAGGAAGAAGC -3′), CDC16-1 siRNA (sequence (I): 5′-GGACGCUUGUAGAGCCU- GATT-3′, (II):5′- UUCAGCUCUACAAGCGUCCTT-3′) and CDC16-2 siRNA (sequence (I): CCGUGGGAUUUCAGGGAAUTT-3′, (II):5′-AUUCCCUGAAAUCCCAC-GGTT-3′) were purchased from ST Pharm (Seoul, Korea). Cells were transfected with siRNA using Lipofectamine™ 2000 (Invitrogen) following the manufacturer’s protocol. The ratio of siRNA versus Lipofectamine reagent was 1:1.15. After transfection, the cells were used for further experiments.

### Transfection with plasmid DNA

Cells were transfected with a plasmid containing YDJC using JetPEI reagent (Polyplus-transfection) according to the manufacturer’s instructions. The universal negative plasmid was employed as the negative control.

### Construction of A549_YDJC_ and A549_YDJCD13A_ cell lines

YDJC or YDJC_D13A_ (deacetylase inactive) overexpressed A549_YDJC_ and A549_YDJCD13A_ were established using a pcDNA3.1 vector containing the YDJC and YDJC_D13A_, and G418 resistance gene as a selection marker.

### Confocal microscopy

Confocal microscopy was performed, as described previously [34]. Cells grown on coverslips and fixed in methanol for 10 min at room temperature were permeabilized with a 10 min wash with 0.1% Triton X-100 at room temperature, followed by several washes in PBS containing 3% BSA. Phospho-K8 and YDJC primary antibody were incubated with the A549 coverslips overnight at 4°C, after which the antibody was removed with four washes with PBS. Species-specific secondary antibodies conjugated with goat anti-mouse IgG antibody (Alexa Fluor 488, 1:500; Molecular Probes) were reacted with the coverslips for 1 h at room temperature followed by four washes with PBS. The final samples were mounted onto slides and visualized using confocal microscopy (Nikon, Tokyo, Japan).

### Immunoprecipitation

Immunoprecipitation (IP) was performed, as described previously [35]. Cell lysates were incubated with rabbit polyclonal anti-YDJC or mouse polyclonal anti-CDC16 antibody overnight at 4°C. Pre-washed protein A/G magnetic beads (Pierce, Rockville, IL, USA) were added to each sample tube and incubated for 1 h at room temperature. Eluted sample volumes of 50 μL were resolved by SDS-PAGE and analyzed by Western blot.

### Prognostic evaluation of YDJC

Prognostic evaluation of YDJC was done as previously described [27, 36].

### Statistical analysis

Data are expressed as the mean ± standard deviation (S.D.) of at least three independent experiments performed in triplicate. Student’s *t*-test was used to analyze the data, according to the following significance levels: ^*^p < 0.05 and ^**^p < 0.01.

## SUPPLEMENTARY MATERIALS FIGURES


