# Supplementary material for: YdjC chitooligosaccharide deacetylase homolog induces keratin reorganization in lung cancer cells: involvement of interaction between YDJC and CDC16
Source: Oncotarget. 2018 May 1;9(33):22915–28. doi: 10.18632/oncotarget.25145 (PMC5955423; doi:10.18632/oncotarget.25145)
Supplement: Supplementary file 1 [file oncotarget-09-22915-s001.pdf]

YdjC chitooligosaccharide deacetylase homolog induces keratin reorganization in lung cancer cells: involvement of interaction between YDJC and CDC16

SUPPLEMENTARY MATERIALS

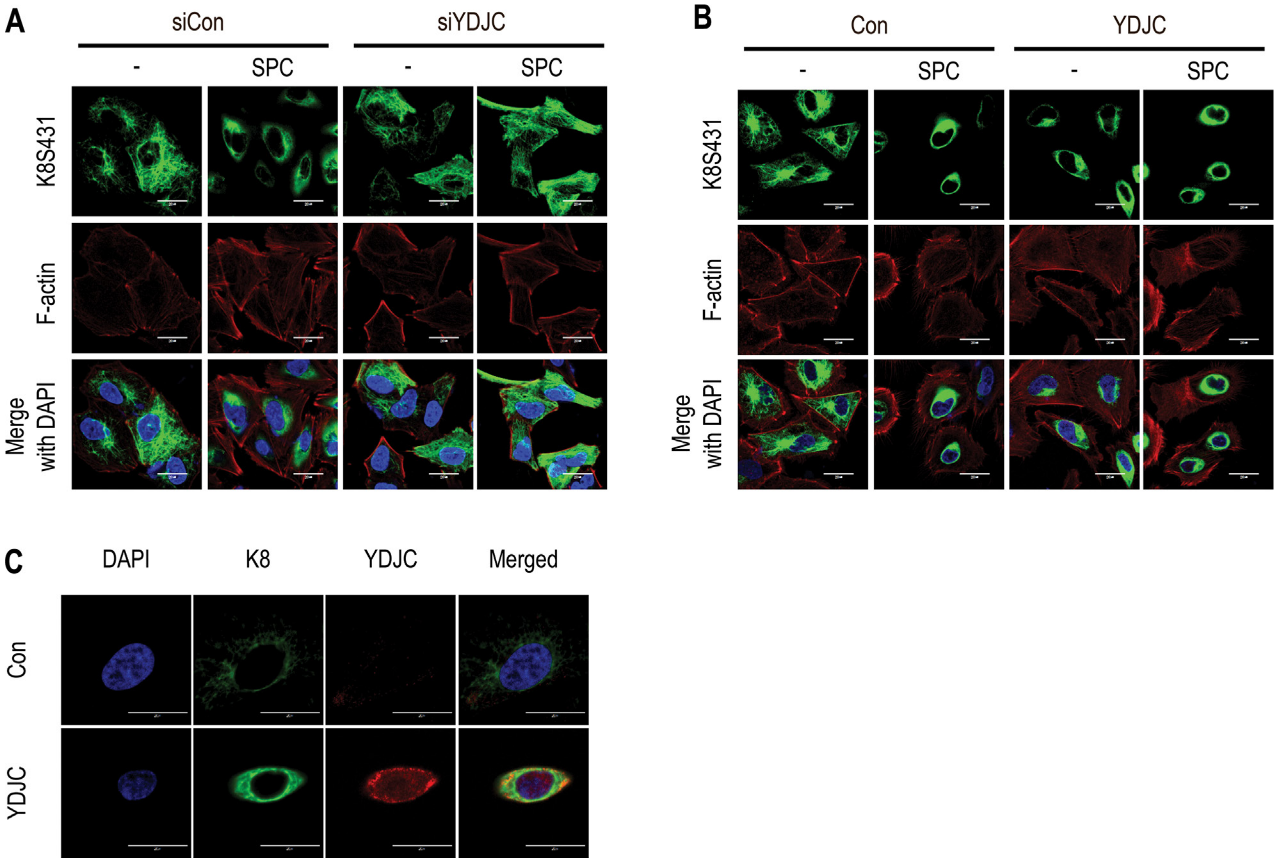

Supplementary Figure 1: Effects of YDJC on K8 reorganization. (**A**) Effect of YDJC siRNA on perinuclear keratin organization in A549 cells stimulated with SPC. A549 cells were stained with anti-K8S431 antibody coupled with FITC-conjugated anti-Rabbit IgG (green) and DAPI (blue). (**B**) Effect of YDJC overexpression on perinuclear keratin organization in A549 cells stimulated with SPC. A549 cells were stained with an anti-K8S431 antibody coupled with FITC-conjugated anti-Rabbit IgG (green) and DAPI (blue). Nuclei were stained with DAPI (blue). Scale bars, 20 µm. (**C**) Confocal microscopic analysis of YDJC and phosphorylation of K8 S431 in A549 cells stimulated with 5 µM SPC for 1 h. Nuclei were stained with DAPI (blue). Scale bars, 10 µm.

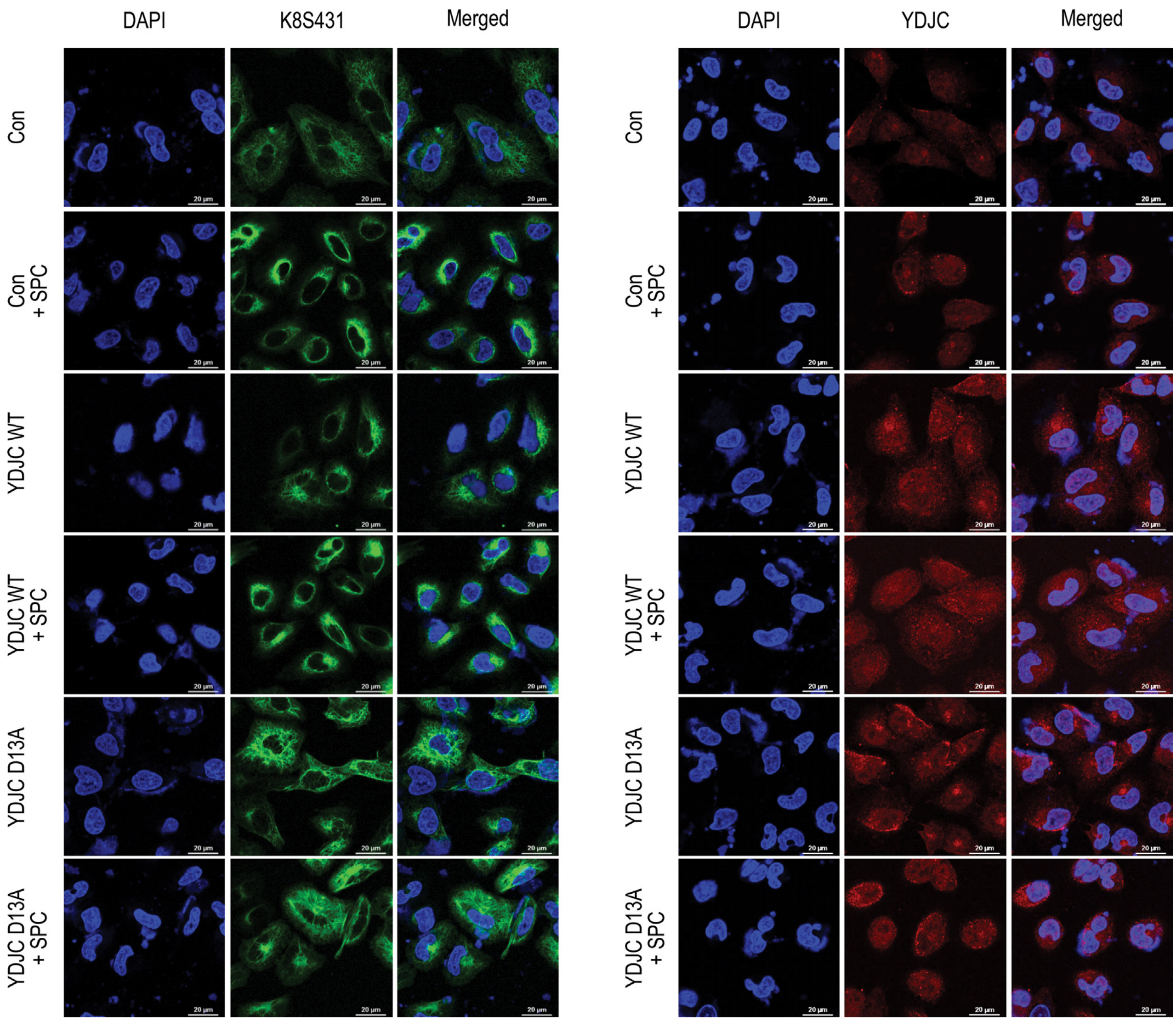

Supplementary Figure 2: Effect of deacetylase activity of YDJC on SPC-induced K8 reorganization. A549 cells were stained with the indicated antibodies. Nuclei were stained with DAPI (blue). Scale bars, 20 µm.

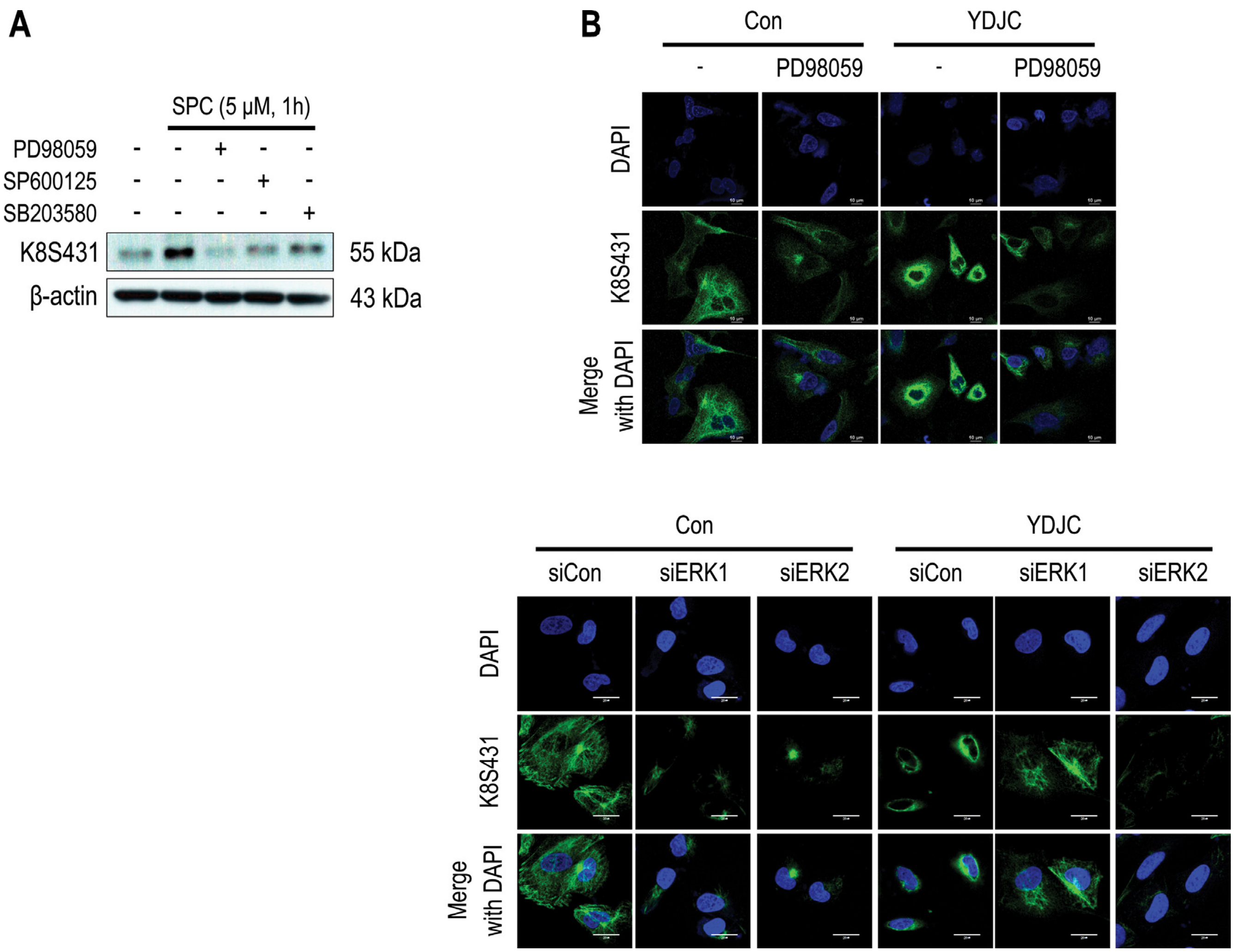

Supplementary Figure 3: Involvement of ERK in the SPC or YDJC-induced K8 phosphorylation and reorganization. (**A**) Effects of MAP kinase inhibitors on SPC-induced K8 phosphorylation in A549 lung cancer cells. A549 cells were treated with or without for 1 hour in the presence of PD98059 (MEK inhibitor, 10 μM), SP600125 (JNK inhibitor, 5 μM), or SB203580 (p38 kinase inhibitor, 10 μM). (**B**) Effect of PD98059 and siRNA of ERK1 or ERK2 on YDJC-induced K8 perinuclear keratin organization. A549 cells were stained with an anti-K8S431 antibody coupled with FITC-conjugated anti-Rabbit IgG (green) and DAPI (blue).

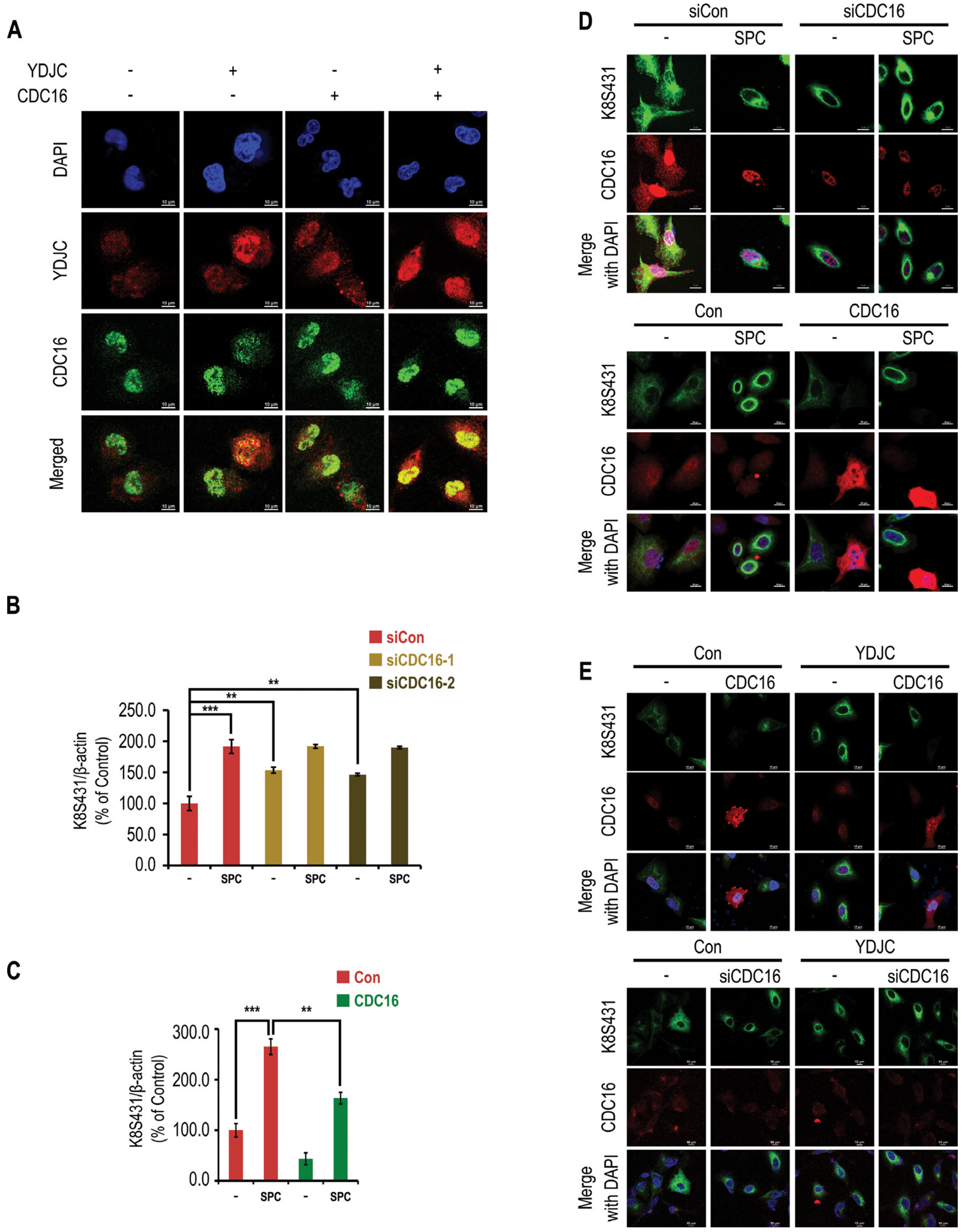

Supplementary Figure 4: Involvement of CDC16 in SPC or YDJC-induced K8 phosphorylation and reorganization. (**A**) Localization of YDJC and CDC16. A549 cells were stained with an anti-YDJC antibody (red) and anti-CDC16 antibody (green) coupled with FITC-conjugated anti-Rabbit IgG, FITC-conjugated anti-Mouse IgG and DAPI (blue). (**B**) Data represent the percentage of K8S431 expression in A549 cells. The data are presented as the mean ± SD from experiments representative of three independent. $^{*}p < 0.05$, $^{**}p < 0.01$, and $^{***}p < 0.001$ compared with the control. (**C**) Data represent the percentage of K8S431 expression in A549 cells. The data are presented as the mean ± SD from experiments representative of three independent. $^{*}p < 0.05$, $^{**}p < 0.01$, and $^{***}p < 0.001$ compared with the control. (**D**) Effect of CDC16 siRNA and CDC16 overexpression on perinuclear K8 reorganization in A549 cells stimulated with SPC. A549 cells were stained with anti-K8S431 antibody coupled with FITC-conjugated anti-Rabbit IgG (green), anti-CDC16 antibody (red) coupled with FITC-conjugated anti-Mouse IgG and DAPI (blue). (**E**) Effect of CDC16 siRNA and CDC16 overexpression on YDJC-induced perinuclear K8 reorganization in A549 cells. A549 cells were stained with anti-K8S431 antibody coupled with FITC-conjugated anti-Rabbit IgG (green), anti-CDC16 antibody (red) coupled with FITC-conjugated anti-Mouse IgG and DAPI (blue). Scale bars, 10 μm.
